# The Self-Regulation of Conformity: Mental Contrasting With Implementation Intentions (MCII)

**DOI:** 10.3389/fpsyg.2021.546178

**Published:** 2021-06-02

**Authors:** Vivica von Weichs, Nora Rebekka Krott, Gabriele Oettingen

**Affiliations:** ^1^Institute of Psychology, Helmut Schmidt University, Hamburg, Germany; ^2^Institute for Interdisciplinary Research on Conflict & Violence (IKG), Bielefeld University, Bielefeld, Germany; ^3^Department of Psychology, New York University, New York City, NY, United States; ^4^University of Hamburg, Hamburg, Germany

**Keywords:** conformity, self-regulation, social influence, mental contrasting with implementation intentions (MCII), computer-mediated communication (CMC)

## Abstract

The self-regulation of conformity has received little attention in previous research. This is surprising because group majorities can exert social strong pressure on people, leading them to overlook the pursuit of their own goals. We investigated if self-regulation by mental contrasting with implementation intentions (MCII) can reduce people’s tendency to conform and facilitate their own goal-pursuit despite deviant majority influence. In a computer-based logical reasoning task, we exposed participants to a conformity manipulation, where we presented bogus diagrams showing the supposedly correct answers of a majority ingroup. Compared to participants who were not given a self-regulation strategy (Studies 1, 2, and 4) or who were in an active control group (Study 3), MCII helped participants to self-regulate conforming behavior in trying to solve the task and to independently solve the logical reasoning task, as indicated by increases in correct answers in the task. The findings suggest that MCII is an effective strategy to regulate people’s tendency to conform and supports them to attain their goal despite deviant majority influence.

## Introduction

Imagine it is quiz night and you and your friends reached the final round. You hear the question, ponder about how to solve the tricky problem, and have an answer in mind of which you think it is the right one. You are not a hundred percent sure, and all your friends insist on another answer. What will you do? Will you keep the answer you had in mind? Or will you conform and go with your friends choosing the other answer although you think it is wrong?

This example shows the conflict of a person who tries to perform correctly but also feels the urge to conform in order to find the right answer or to prevent exclusion from the group. Conformity has been defined as the tendency to change one’s attitudes or behaviors to match the responses of others (Cialdini and Goldstein, 2004; Kim and Hommel, 2015). People adapt their attitudes and behaviors to those of others when facing uncertain situations, thereby using other people’s judgments as their source of information about the “real” value of the object under consideration (*informational influence*; Deutsch and Gerard, 1955; Cialdini, 1993; see also Sherif, 1936; Festinger, 1954; Erb and Bohner, 2007). People also adapt their attitudes and behaviors to those of others in order to obtain social approval or to meet other people’s expectations, thereby avoiding sanctions or even punishment for being deviant (*normative influence*; Deutsch and Gerard, 1955; Cialdini and Goldstein, 2004; Baumeister et al., 2007). And they sometimes do so, even if they know that these behaviors are incorrect (Asch, 1955). Relatedly, people are more likely to conform to majority groups when they are viewed as an in-group (i.e., shared social identity; *self-categorization theory*; Turner, 1985, 1991) than when they are viewed as an out-group (i.e., no shared social identity; Turner, 1991; David and Turner, 1996, 2001; Crano, 2001; Erb and Bohner, 2007).

### Consequences of Conformity

Conformity is an important force that keeps groups together and facilitates communication (e.g., Argyle, 1957; Hollander, 1958; LaTour and Manrai, 1989; Bond and Smith, 1996). However, the pressure to conform can also have detrimental effects for the individual. It can seduce people to concur with false information or to engage in risky behaviors (Asch, 1951, 1956; Santor et al., 2000; Lynn and Snyder, 2002; Gardner and Steinberg, 2005; Zhou et al., 2009). Conformity pressure may not only arise in the physical presence of others. Even in computer-mediated contexts people may feel the urge to follow the behavior of others (e.g., McKenna and Bargh, 1998; Postmes et al., 1999; Postmes and Spears, 2002; Riva, 2002; Lee, 2006; Cinnirella and Green, 2007; Lee et al., 2008; Schlosser, 2009; Laporte et al., 2010; Park and Feinberg, 2010; Bak and Keßler, 2012; Rosander and Eriksson, 2012; Anderson et al., 2014).

Conformity with the majority group can also cause people to disregard the successful pursuit of their own individual wishes or goals (e.g., Deutsch and Gerard, 1955; Asch, 1956). However, little research so far has focused on strategies aimed at reducing conforming behavior that may impede one’s own goal attainment. In the present studies, we tested whether the self-regulation strategy of mental contrasting with implementation intentions (MCII) would help people to regulate their tendency to conform, and thus support them in attaining their goals despite deviant majority influence.

### Regulation of Conformity

Research on how to attenuate conformity points out that prompts protecting people’s self-esteem are effective in reducing susceptibility to other people’s opinions and perceptions (e.g., writing about a positive, stable inner trait leads to less conforming behavior, Arndt et al., 2002; see also Imhoff and Erb, 2009). While strategies affirming people’s self-esteem may be successful in reducing conforming behavior, they require an external prompt, such as feedback from others about one’s current behavior. But how can people protect themselves from submitting to majority opinions when those external prompts (e.g., by the researcher, educator, or any other authority) are not available or are not desired? Such regulating conformity is particularly difficult because people often conform without consciously realizing it and may even deny having been influenced by others (e.g., Chartrand and Bargh, 1999; Hornsey and Jetten, 2004, 2005; Bargh et al., 2012). Accordingly, people often fail to identify their tendency to conform to the majority opinion as an obstacle that holds them back from attaining their personal goals.

We introduce MCII to help people reduce their tendency to act in a conform way as MCII has been found to help people attain their goals even if they were not aware of the obstacle once it occurs. That is, MCII is a conscious imagery technique with non-conscious cognitive and motivational processes that enables people to surmount their obstacles and achieve behavior change outside of awareness (Kappes et al., 2012, 2013; Kappes and Oettingen, 2014). Applied to the present context, MCII should support people to strive for their individual goals in the face of a deviant majority even if they might not be aware of being tempted to give the majority’s answer once the majority’s answers are presented. Further, in contrast to previous studies, where participants wrote about a stable inner trait within a specific situation, MCII is – once it is learned by the individual – applicable to every situation in which people may be biased by the social influence of other people.

### Mental Contrasting With Implementation Intentions (MCII)

#### Mental Contrasting

Fantasy realization theory (Oettingen, 2000, 2012, 2014) identifies mental contrasting as a self-regulation strategy that fosters effort and performance toward a desired and feasible future. During mental contrasting, people first positively fantasize about a desired future (e.g., finding the correct solution for a tricky problem) and then imagine the obstacle in the present reality (e.g., the urge to give in to your friends insisting on a different solution than yours). Imagining reaching the desired future followed by imagining the obstacle reveals that achieving one’s desired future requires acting on the obstacle of present reality (e.g., be daring, speak up). When the obstacle is surmountable, mental contrasting strengthens effort toward the desired future (e.g., to independently solve the tricky problem). Mental contrasting has been shown to foster effort and performance across different life domains, ages, and cultures, for short-term as well as long-term goals, and for different indicators of goal attainment (e.g., cognitive, emotional, and behavior; Oettingen et al., 2001; review by Oettingen, 2012; Oettingen and Sevincer, 2018).

With respect to non-conscious cognitive processes, mental contrasting helps people to detect obstacles of present reality standing in the way of attaining the desired future. It also changes the implicit meaning of reality as an obstacle that needs to be overcome. Further, mental contrasting strengthens the associative links between the desired future and the obstacle of reality as well as between the obstacle and the instrumental means to overcome the obstacle. Those cognitive processes mediate mental contrasting effects on effort and performance (Kappes et al., 2012, 2013; Kappes and Oettingen, 2014).

Fantasy realization theory identifies another mode of thought about the desired future: Reverse contrasting. When people engage in reverse contrasting, they elaborate on the same content as in mental contrasting, but in reverse order (i.e., the present reality followed by the desired future). Accordingly, the relational construct of reality standing in the way of the desired future is not activated and the reality is not interpreted as an obstacle (Kappes et al., 2013). Therefore, reverse contrasting fails to instigate behavior change (review by Oettingen, 2012).

#### Implementation Intentions

Mental contrasting has been combined with implementation intentions (MCII; Adriaanse et al., 2010; Kirk et al., 2013; Oettingen et al., 2015; review by Oettingen, 2012). Implementation intentions (Gollwitzer, 1993, 1999, 2014) are plans that prepare for goal pursuit by linking a predetermined action to a certain situation (“If situation X occurs, then I will perform behavior Y.”), thereby explicitly specifying when, where, and how one wants to act toward realizing one’s goal. They are differentiated from sheer goal intentions that come in the form of: “I want to perform behavior Y!”.

Implementation intentions (vs. sheer goal intentions) foster goal pursuit by making the mental representation of the situation specified in the if-part highly accessible (Gollwitzer, 1999) and by strengthening the associative links between this situation and the instrumental behavior specified in the then-part (Webb and Sheeran, 2007, 2008; review by Gollwitzer, 2014). Those associative links ensure that mental representations of the specified behavior are activated whenever the specified situation is encountered, leading to features of automaticity in goal directed behavior (i.e., immediacy, efficiency, no need of conscious intention, and autonomy; *strategic automaticity*, Gollwitzer and Brandstätter, 1997; Gollwitzer, 1999).

Implementation intentions (vs. goal intentions) have been shown to be effective across various life domains (reviews by Gollwitzer, 1999, 2014; Gollwitzer and Sheeran, 2006) but only when the conditions of goal pursuit are met, that is, when people are fully determined to reach their goal, and when the critical situation (if-part) as well as the goal-directed action (then-part) are clearly specified. Mental contrasting meets all three requirements. Specifically, it creates a strong determination or goal commitment, it highlights the critical situation (i.e., the obstacle of present reality), and it strengthens the implicit associative links between the obstacle and the behavior to overcome the obstacle (Kappes et al., 2012). Indeed, mental contrasting with implementation intentions (MCII) has been found to be more effective than either of the strategies alone (Adriaanse et al., 2010; Kirk et al., 2013).

### The Present Research: Self-Regulation of Conformity Using MCII

Mental contrasting with implementation intentions captures three significant aspects relating to conforming behavior. First, by promoting the identification of the relevant obstacle (Kappes et al., 2013), MCII should allow people to anticipate situations where they might be giving in to the majority’s influence to then automatically resist the influence once it occurs. Second, by strengthening the associative link between the obstacle and the instrumental means to overcome the obstacle (Kappes et al., 2012), MCII should help people to carry out the pre-specified behaviors that help them overcome the obstacle (e.g., ignoring other people’s behavior), and third, by forming implementation intentions people should build particularly strong associative links between the obstacle and the behavior to overcome the obstacle. Thus, people should tightly stick to the goal-directed behavior they had previously decided upon once the obstacle occurs.

### Pilot Study: Establishing the Paradigm

Because in the present research we recruited participants on Amazon Mechanical Turk (MTurk), we were able to introduce a respective shared social identity and an in-group context (i.e., the majority of MTurk participants). Specifically, we made social identity salient by introducing the community of MTurk participants as an in-group, whose results were allegedly compared to those of an out-group (i.e., bankers). Despite that identity of an MTurk worker is most likely not the central identity of our participants, making the in-group context of being an MTurk worker salient to our participants may additionally increase their tendency to conform to the behaviors of the group of MTurk participants.

In all studies, we report all measures, manipulations and exclusions. All sample sizes were determined before any data analysis. We first developed an on-line paradigm to induce conformity following a study design by Rosander and Eriksson (2012). We asked participants to answer logical reasoning items, which were taken from the *Standard Progressive Matrices* (Raven, 1965). In a pre-test with 50 participants, we assessed the difficulty level of eleven Raven matrices. Consulting the percentage of correct answers for each item, we chose eight matrices of varying difficulty, from the easiest item (96% of the participants answered correctly) to the most difficult item (40% of the participants answered correctly).

**FIGURE 1:**
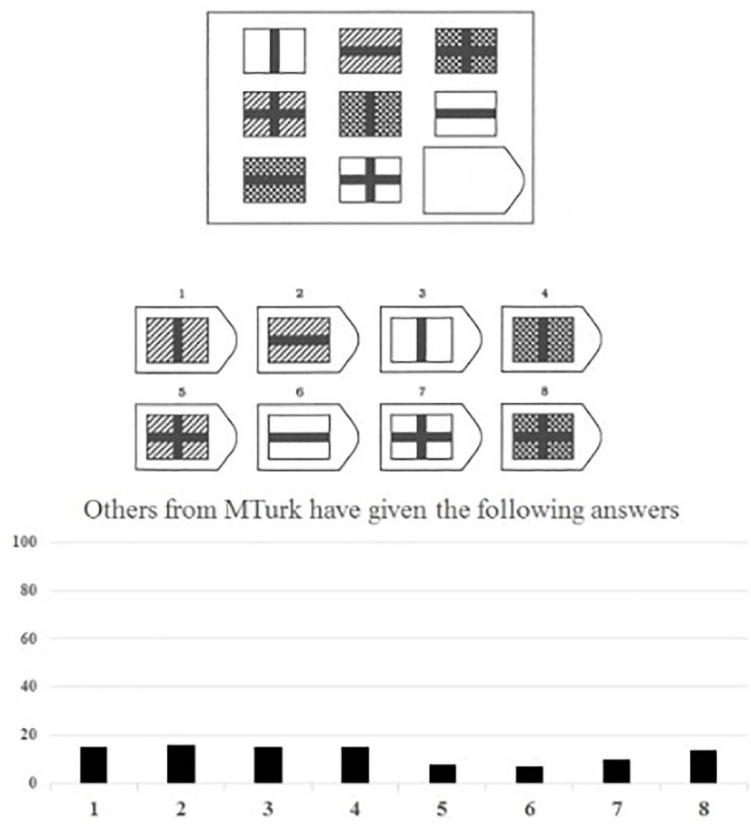
Example of logical reasoning item (Item 4) with the fabricated diagram shown to the control condition. The Correct answer for the logical reasoning item (Item 4) is no 1.

**FIGURE 2:**
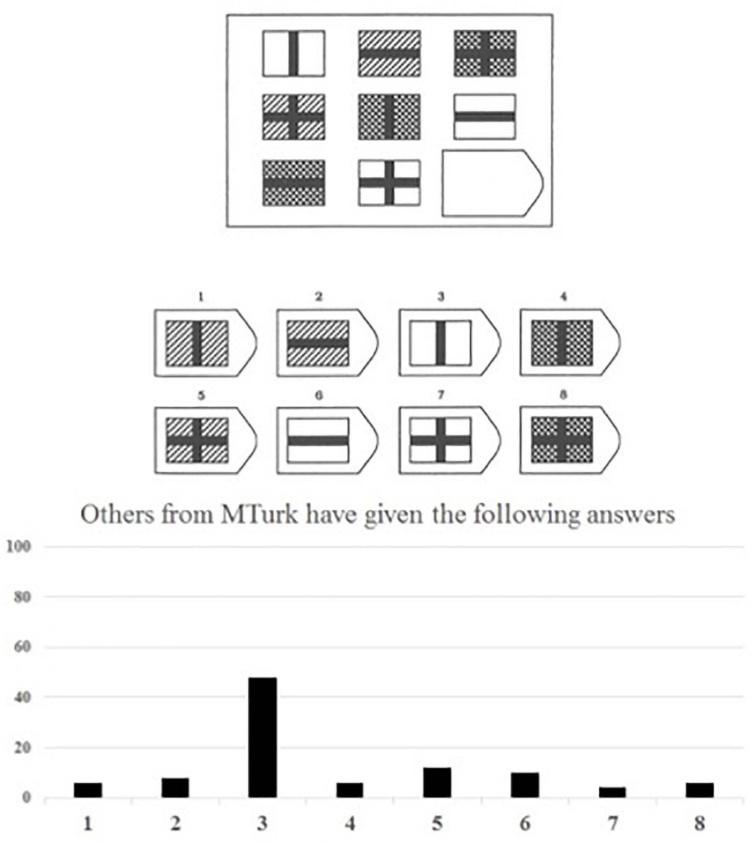
Example of logical reasoning item (Item 4) with the fabricated diagram shown to the conformity condition.

In this Pilot Study, we randomly assigned participants to a conformity condition or a control condition. While solving the Raven matrices, all participants were presented with diagrams showing how other MTurk participants had supposedly answered so far. Participants in the control group saw equally distributed answers for each item (Figure 1), participants in the conformity group saw that a majority choosing one particular incorrect answer (Figure 2)^1^. In line with the results of Rosander and Eriksson (2012), participants in the conformity group (vs. control group) chose more often those incorrect answers that were indicated by the supposed majority (see the Supplementary Materials for methods and results; see also Riess, 2017).

## Study 1: MCII vs. No Self-Regulation

In Study 1, we aimed to replicate findings from the Pilot Study, i.e., the induction of conformity within our computer-based paradigm. Specifically, we expected *a priori* contrasts to show that participants in the conformity group would more often choose those incorrect answers that were indicated by the supposed majority compared to participants in the control group. Second, and most importantly, we aimed to investigate whether MCII is an effective strategy to support people in regulating their tendency to conform and to help them reach their goal of independently solving the logical reasoning task. Specifically, we hypothesized that participants in the conformity condition using the strategy of MCII (vs. no self-regulation strategy) would give fewer answers complying with the incorrect answers of the supposed majority and give a greater number of correct answers.

### Method Study 1

#### Participants and Power Analysis

Based on previous research regarding the induction of conformity in the context of CMC, we assumed a potential study drop out of up to 10% of the participants. We recruited 157 participants. A total of 20 participants were excluded because they indicated suspicion about the conformity manipulation (*n* = 15, for example, one participant wrote “I think that the other Mturk worker responses weren’t real.”) or did not follow the instruction of the self-regulation strategies (*n* = 5; for example, one participant wrote “Jumping up and down” when asked to elaborate on the outcome and obstacle)^2^.

All participants were recruited on MTurk and received $2.00 for their participation. The final sample consisted of 137 participants, of which 70.1% were women. The participants’ ages ranged between 19 and 75 years with a mean of 39.46 years (*SD* = 13.89). All participants were randomly assigned to either a conformity or a control condition. Additionally, participants either received MCII or no self-regulation strategy, resulting in four conditions (see Table 1). A sensitivity power-analysis indicated that with 66 participants (within the two conformity conditions) and 71 participants (within the two control conditions) we would have 90% power to find, in each case, a large effect (*d* = 0.81 and *d* = 0.79).

**TABLE 1:** Number of participants per condition.

Study	*Conformity*			*Control*		
	*No SRS*	*MCII*	*RC*	*No SRS*	*MCII*	*RC*
Study 1	31	35	–	41	30	–
Study 2	50	46	–	53	46	–
Study 3	53	44	45	43	52	44
Study 4	44	40	–	46	47	–

*Participants were randomly assigned to the conditions. No SRS, no self-regulations strategy; MCII, Mental Contrasting with Implementation Intention; RC, reverse contrasting.*

#### Procedure and Materials

All participants completed the study online. Prior to their participation, they were informed about the procedure of the study and completed the consent form. After reading the cover story, participants were asked about their expectations, incentive and commitment to independently solve the upcoming logical reasoning task. They were then randomly assigned to the experimental conditions. Thereafter, participants completed the logical reasoning task and were finally asked for some demographic data. After completion of the study, all participants were thanked, debriefed and received credit for their participation in the survey.

##### Social identification

We presented participants the same cover story as in the pilot study, i.e., explaining that the survey was designed to compare cognitive abilities of people who occasionally deal with social psychology experiments (e.g., MTurk participants) and people who deal with economic problems only (e.g., bankers). We also asked participants to what extent they identified with the group of MTurk workers (see Supplementary Materials for more details on social identification).

##### Expectations, incentive, and commitment

After the cover story we presented participants an example of the logical-reasoning task. We asked them to think about independently solving the upcoming logical reasoning task: “Think about how nice it would be if you independently solved all of the following tasks successfully and could say to yourself: “Yes! I did it right!” Thereafter, we assessed participants’ incentive value (i.e., “How important is it to you that you will independently solve all of the following tasks successfully?”), expectations of success (i.e., “How likely do you think it is that you will independently solve all of the following tasks successfully?”) and commitment (i.e., “How disappointed would you feel if you did not independently solve all of the following tasks successfully?”). The Likert scales reached from 1 (*not at all*) to 7 (*very*), with high scores indicating high expectations, incentive, and commitment.

#### Manipulation of the Self-Regulation Strategy

Participants were randomly assigned to one of two experimental conditions. In the MCII condition, they were asked to write down one positive aspect they associate with independently solving the upcoming logical reasoning task. Participants read: “What would be the best thing if you independently solved all of the following tasks successfully and could say to yourself: “Yes! I did it right!”? What would be the most wonderful thing about it?” Participants wrote, for example, that they would feel good about themselves having the ability to independently complete the tasks successfully and that they would feel smart because they did the tasks by themselves. For example, one participant named “I would feel accomplished.” Then, participants were asked to imagine their positive aspect. Participants read: “Now take a moment and imagine your best outcome. Imagine things fully. Please write thoughts and images down.” For example, the same participant wrote “I would be proud of myself for being able to answer all of the questions on my own. It would be an accomplishment to be proud of.” Next, we introduced participants to the obstacle that might hinder them from independently succeeding in solving the logical reasoning task and asked them to imagine it. Participants read:

Sometimes things don’t work out as we would like them to. People tend to follow other people’s behavior when they are unsure of how to act. This can often lead to mistakes. Imagine yourself following the behavior of other people when solving the cognitive tasks. Imagine things fully. Please write your thoughts and images down.

Here, participants named, for example, that, especially when being insecure about how to behave, they would look at what other people are doing and emulate their actions even though they would be disappointed at themselves for not solving the test by themselves. For example, the participant cited above named “I would observe how others acted and try to mimic their behavior if they appeared to be answering the questions successfully.” Finally, participants were shown a plan, which we asked them to repeat, write down one more time, and remember whenever they would feel that they followed other people’s behavior during the task: “If I feel that I follow other people’s behavior, then I will tell myself: Ignore them!”

In the no self-regulation strategy condition, participants were immediately directed to the logical reasoning task after they had indicated their expectations, incentive and commitment to independently solve the logical reasoning task.

### Results and Discussion Study 1

#### Expectations, Incentive, and Commitment

Mean values for expectations [*M* = 5.12, *SD* = 1.49; *t*(136) = 8.77, *p* < 0.001], incentive value [*M* = 6.07, *SD* = 1.10; *t*(136) = 5.86, *p* < 0.001], and for commitment [*M* = 4.84, *SD* = 1.68; *t*(136) = 22.01, *p* < 0.001] were above the midpoints of the scales. There were no significant differences between the conditions regarding the items (all *p*’s > 0.5, see Supplementary Materials).

#### Conformity on the Logical Reasoning Task

Identical to the Pilot Study, participants in the conformity condition were able to conform zero to five times. Again, we quantified conformity as the difference between the number of answers that agreed with the supposed majority answers for participants in the conformity condition and the number of the same incorrect answers for participants in the control condition.

We conducted a two-way ANOVA with conformity (*conformity* vs. *control*) and condition (*MCII* vs. *no self-regulation*) as between-subject factors and the number of conform answers on the task as dependent variable. There was a significant main effect of conformity, *F*(1,133) = 16.34, *p* < 0.001, *d* = 0.69, indicating that participants who were exposed to false majority answers more often chose the respective incorrect answers compared to participants who were not exposed to false majority answers. There also was a significant main effect of condition, *F*(1,133) = 5.04, *p* = 0.026, *d* = 0.38, with participants adopting MCII choosing the incorrect answer indicated by the majority less often compared to participants adopting no self-regulation strategy. The interaction between conformity and condition did not reach significance, *F*(1,133) = 2.75, *p* = 0.100, *d* = 0.20.

However, we exclusively aimed to investigate our first *a priori* hypothesis, i.e., the induction of conformity within the paradigm. We conducted planned contrasts (according to Furr and Rosenthal, 2003) for both conditions adopting no self-regulation strategy (*control no self-regulation* vs. *conformity no self-regulation*), thereby assigning a weight of 0 to both conditions using a self-regulation strategy. In line with our hypothesis, participants in the *conformity no self-regulation* condition more often chose the incorrect answer indicated by the supposed majority (*M* = 1.89, *SD* = 1.51) compared to participants in the *control no self-regulation* condition [*M* = 0.78, *SD* = 0.85), *t*(51.73) = 3.84, *p* < 0.001, *d* = 0.93]. Thus, we successfully induced conformity within the computer-based paradigm.

**FIGURE 3:**
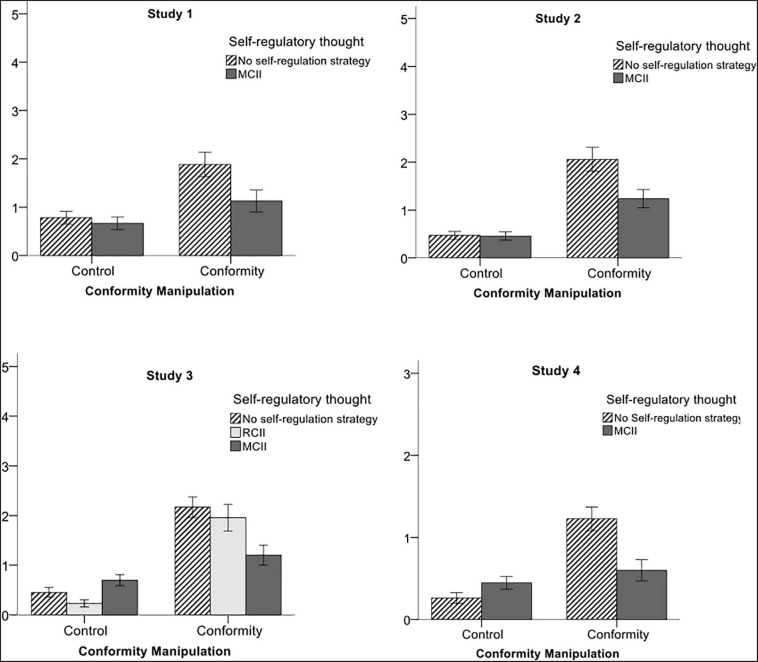
Mean number of conform answers (dependent variable) for all conditions. Standard errors are represented by the error bars attached to each column.

To test our second hypothesis, i.e., participants adopting MCII chose less often the incorrect answers indicated by the supposed majority, we conducted planned contrasts between both conformity conditions (*conformity MCII* vs. *conformity no self-regulation*), assigning a weight of 0 to both control conditions. In line with our hypothesis, participants in the *conformity MCII* condition chose the incorrect answer indicated by the supposed majority less often (*M* = 1.13, *SD* = 1.23) than participants in the *conformity NSR* condition (*M* = 1.89, *SD* = 1.51), *t*(63.78) = 2.22, *p* = 0.030, *d* = 0.55 (Figure 3). Specifically, participants in the *conformity no self-regulation* condition went along with the false majority 37.8% of the time; this effect was reduced to 22.6% in participants in the *conformity MCII* condition^3^.

#### Self-Regulation of Correct Behavior

We performed our analysis of correct behavior on the five critical items. Thus, participants could obtain a maximum of five correct answers on the task. We conducted a two-way ANOVA with conformity (*conformity* vs. *control*) and condition (*MCII* vs. *no self-regulation*) as between-subject factors and the number of correct answers on the task as dependent variable. There was no significant main effect of conformity, *F*(1,133) = 2.11, *p* = 0.149, *d* = 0.26. There was, however, a significant main effect of condition, *F*(1,133) = 7.27, *p* = 0.008, *d* = 0.46, indicating that participants who used MCII answered more items correctly compared with participants who did not use a self-regulation strategy. The interaction between conformity and condition was not significant, *F*(1,133) = 0.03, *p* = 0.856, *d* = 0.00.

**FIGURE 4:**
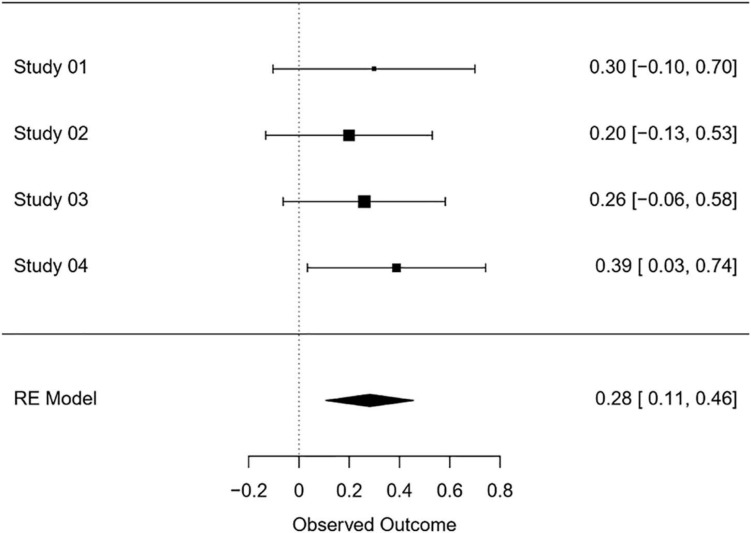
Meta-analysis of the four studies. Forest plot, random effects model were calculated for the number of correct answers on the task as dependent variable, including condition as independent variable.

However, to investigate our *a priori* hypothesis, i.e., participants in the conformity conditions would increase the number of correct answers on the task, we conducted planned contrasts for both conformity conditions (*conformity MCII* vs. *conformity no self-regulation*) on the number of correctly solved items, assigning a weight of 0 to both control conditions. There was a marginally significant difference between the two conditions; participants in the *conformity MCII* condition tended to give more correct answers on the task (*M* = 3.06, *SD* = 1.73) compared to those in the *conformity no self-regulation* condition (*M* = 2.34, *SD* = 1.57), *t*(133) = 1.76, *p* = 0.082, *d* = 0.30 (Figure 4).

To conclude, even though the interaction effects did not reach significance, our main hypotheses focusing on comparing the effect of MCII (vs. not self-regulation) within the two conformity conditions were tentatively supported. Participants showed conformity in a computer mediated context and the findings speak for the observation that participants regulated their tendency to conform when engaging in MCII compared to participants engaging in no self-regulation strategy. Further, participants in the conformity condition tended to give more correct answers on the logical reasoning task when engaging in MCII compared to participants engaging in no self-regulation strategy. This marginally significant difference might indicate that MCII may have supported participants in attaining their goal of independently solving the task. The greater number of correct answers may even suggest that participants did not just blindly follow the instruction of ignoring other people’s behavior by choosing any other option than the majority. Rather, participants seemed to have more successfully completed the task on their own, though we need to interpret this result with caution as the interaction effect between conformity and the self-regulation condition was not significant.

In Study 2, we aimed to replicate our findings using a slightly modified version of the logical reasoning task. While we used logical reasoning items with increasing difficulty in the first study, we now used items with a medium level of difficulty (i.e., those items for which approximately 60–70% of the participants found the correct answer when working uninfluenced on the items), since they appeared most appropriate for the needs of our study. These items were easy enough for participants to answer them correctly when being focused, but still included some complexities so that participants would potentially turn over to the behavior of the majority to seek guidance for their choices.

## Study 2: MCII vs. No Self-Regulation (Direct Replication)

We aimed to directly replicate the findings from Study 1.

### Method Study 2

#### Participants and Power Analysis

We recruited 206 participants. A total of 11 participants were excluded because they indicated suspicion about the conformity manipulation (*n* = 7; for example, one participant wrote “The Other MTurk results were clearly fictitious!”) or did not follow the instruction of the manipulation of self-regulatory thought (*n* = 4; for example, one participant wrote “Thank you for life, and all the little ups and downs that make it worth living.” instead noting an *if-then* plan). Thus, we included 195 participants (65.1% women) with a mean age of 37.06 years (*SD* = 13.09) in the analysis and randomly assigned them to one of four conditions (see Table 1). A sensitivity power-analysis indicated that with 96 participants (within the two conformity conditions) and 99 participants (within the two control conditions) we would have 90% power to find, in each case, a medium to large effect (*d* = 0.67 and *d* = 0.66).

#### Procedure and Materials

The procedure and materials of Study 2 were identical to those used in Study 1, except for two logical reasoning items. We replaced the easiest as well as the most difficult item with two items of medium difficulty, thereby creating a consistent level of medium difficulty throughout the experiment.

### Results and Discussion Study 2

#### Expectations, Incentive, and Commitment

Mean values for expectations [*M* = 5.27, *SD* = 1.38; *t*(194) = 12.81, *p* < 0.001], incentive [*M* = 6.08, *SD* = 1.03; *t*(194) = 28.16, *p* < 0.001], and commitment [*M* = 4.64, *SD* = 1.60; *t*(194) = 5.54, *p* < 0.001] were above the scale midpoints. There were no significant differences in expectations, incentive, or commitment between the four conditions (all *p*’s > 0.5, see Supplementary Materials).

#### Conformity on the Logical Reasoning Task

The two-way ANOVA with conformity (*conformity* vs. *control*) and condition (*MCII* vs. *no self-regulation*) as between-subject factors and the number of conform answers on the task as dependent variable showed a significant main effect of conformity, *F*(1,191) = 49.05, *p* < 0.001, *d* = 1.02, as well as a significant main effect of condition, *F*(1,191) = 6.09, *p* = 0.014, *d* = 0.36. The interaction between conformity and condition was also significant, *F*(1,191) = 5.66, *p* = 0.018, *d* = 0.36. As in Study 1, focused planned contrasts showed that participants in the *conformity no self-regulation* condition more often chose the incorrect answers indicated by the supposed majority (*M* = 2.06, *SD* = 1.78) compared to participants in the *control no self-regulation* condition [*M* = 0.47, *SD* = 0.61 *t*(59.72) = 5.99, *p* < 0.001, *d* = 1.21]. Thus, we successfully induced conformity.

With regard to the regulation of conformity, participants in the *conformity MCII* condition gave fewer incorrect answers in accordance with the supposed majority (*M* = 1.24, *SD* = 1.30) compared to participants in the *conformity no self-regulation* condition [*M* = 2.06, *SD* = 1.78 *t*(89.65) = 2.59, *p* = 0.011, *d* = 0.52] (Figure 3). In total, participants in the conformity condition adopting no self-regulation strategy went along with the supposed majority 41.2% of the time; this effect was reduced to 24.8% in participants adopting MCII.

See the Supplementary Materials for our results regarding social identification with the majority (i.e., the group of MTurk participants) and the analysis of conformity as a mediator of the effect of condition (MCII vs. no self-regulation strategy) on social identification with the majority.

#### Self-Regulation of Correct Behavior

We conducted a two-way ANOVA with conformity (*conformity* vs. *control*) and condition (*MCII* vs. *no self-regulation*) as between-subject factors and the number of correct answers on the task as dependent variable. There was no significant main effect of conformity, *F*(1,191) = 0.21, *p* = 0.646, *d* = 0.06. There was, however, a significant main effect of condition, *F*(1,191) = 7.29, *p* = 0.008, *d* = 0.38, indicating that participants who used MCII answered more items correctly compared with participants who did not use a self-regulation strategy. The interaction between conformity and condition was not significant, *F*(1,191) = 0.43, *p* = 0.515, *d* = 0.08. Similar to Study 1, though the interaction effect was not significant, *a priori* contrasts showed that participants in the *conformity MCII* condition gave more correct answers on the task (*M* = 2.98, *SD* = 1.53) compared to participants in the *conformity no self-regulation* condition (*M* = 2.22, *SD* = 1.89), *t*(191) = 2.35, *p* = 0.020, *d* = 0.35 (Figure 4).

In total, we were able to replicate the findings from the first study. However, one could argue that the findings on the regulation of conforming behavior and the tentative observation on the improvement of correct behavior on the task were driven by the information provided in the MCII condition, compared to the control condition. Specifically, while participants engaging in MCII received a clue concerning the obstacle of being influenced by other people’s behavior (i.e., “People tend to follow other people’s behavior when they are unsure of how to act”), participants in the control condition received no such information. In Study 3, we aimed to demonstrate that it is not the provided content but rather *how* people think about that content that causes them to regulate their tendency to conform. We included an additional control condition of reverse contrasting. In the reverse contrasting condition, participants elaborated on the same content as participants in the MCII condition, but in reverse order (i.e., the obstacle of present reality followed by the best outcome of attaining their goal). Further, they formulated an *if-then* plan in the form of *if* (outcome)…*then* (emotion).

## Study 3: MCII vs. Reverse Contrasting vs. No Self-Regulation

Using the same paradigm as in the previous studies, we aimed to further support our hypotheses that participants in the conformity condition adopting MCII (vs. respective reverse contrasting or no self-regulation strategy) would reduce the number of conform and increase the number of correct answers on the task.

### Method Study 3

#### Participants and Power Analysis

We recruited 300 participants. We excluded 20 participants from the analysis because they indicated suspicion about the conformity manipulation (*n* = 12; for example, one participant wrote “I do not believe the responses given by other workers were real”) or did not follow the instruction of the manipulation of self-regulatory thought (*n* = 8; for example, one participant wrote “no” instead of elaborating on the outcome). All participants were recruited on MTurk and received $2.00 for their participation. We included 280 participants in the analyses, of which 63.2% were women with a mean age of 38.54 years (*SD* = 13.94). All participants were randomly assigned to one of six conditions (see Table 1). A sensitivity power-analysis indicated that with 142 participants (within the two conformity conditions) and 139 participants (within the two control conditions) we would have 90% power to find, in each case, a medium effect (*d* = 0.59 and *d* = 0.57).

#### Procedure and Materials

The procedure and materials were identical to those used in Study 2. In the reverse contrasting condition, participants elaborated on the same content as participants in the mental contrasting condition. However, they first imagined the obstacle of being influenced by other people’s behavior, and thereafter named and imagined the best outcome of successfully solving the task. Finally, we asked participants to make a mute plan (i.e., “If I solve all following tasks successfully, then I will feel great!”), which they were requested to repeat.

### Results and Discussion Study 3

#### Expectations, Incentive, and Commitment

Mean values for expectations [*M* = 4.99, *SD* = 1.40; *t*(279) = 11.82, *p* < 0.001], incentive value [*M* = 5.85, *SD* = 1.22; *t*(279) = 25.35, *p* < 0.001] and commitment [*M* = 4.43, *SD* = 1.02; *t*(279) = 4.13, *p* < 0.001] were above the scale midpoints. There were no significant differences between the conditions concerning these items (all *p*’s > 0.5, see Supplementary Materials).

#### Conformity on the Logical Reasoning Task

We conducted a two-way ANOVA with conformity (*conformity* vs. *control*) and condition (*MCII* vs. *RC* vs. *no self-regulation*) as between-subject factors and the number of conform answers on the task as dependent variable. There was a significant main effect of conformity, *F*(1,274) = 84.89, *p* < 0.001, *d* = 1.12, whereas the main effect of condition did not reach significance, *F*(2,274) = 2.21, *p* = 0.111, *d* = 0.26. The interaction between conformity and condition was significant, *F*(2,274) = 7.79, *p* = 0.001, *d* = 0.48. Testing our *a priori* hypothesis, we consulted focused planned contrasts, assigning the weight 0 to the condition adopting a self-regulation strategy. Participants in the *conformity no self-regulation* condition chose the incorrect answers indicated by the supposed majority significantly more often (*M* = 2.17, *SD* = 1.50) compared to participants in the *control no self-regulation* condition (*M* = 0.45, *SD* = 0.70), *t*(74.27) = 7.52, *p* < 0.001, *d* = 1.46.

Regarding the self-regulation of conformity, planned contrasts showed that participants in the *conformity MCII* condition chose the incorrect answers indicated by the supposed majority less often (*M* = 1.20, *SD* = 1.32) compared to participants in both the *conformity RC* condition (*M* = 1.96, *SD* = 1.78), and the *conformity no self-regulation* condition (*M* = 2.17, *SD* = 1.50 *t*(100.71) = 3.29, *p* < 0.001, *d* = 0.65 (Figure 3). Specifically, participants who engaged in reverse contrasting or no self-regulation strategy went along with the false majority approximately 41.3% of the time, whereas this effect was reduced to 24.0% in participants engaging in MCII. See the Supplementary Materials for our results on the mediating role of conformity for the effect of condition [MCII vs. reverse contrasting (RC) or no self-regulation strategy] on social identification with the majority.

#### Self-Regulation of Correct Behavior

Identical to Studies 1 and 2, we performed our analysis of correct behavior on the five critical items. Thus, participants could obtain a maximum of five correct answers on the task. We conducted a two-way ANOVA with conformity (*conformity* vs. *control*) and condition (*MCII* vs. *RC* vs. *no self-regulation*) as between-subject factors and the number of correct answers on the task as dependent variable. Again, there was a significant main effect of conformity, *F*(1,274) = 14.65, *p* < 0.001, *d* = 0.46, as well as a marginal significant main effect of condition, *F*(2,274) = 2.85, *p* = 0.059, *d* = 0.28. The interaction between conformity and condition was not significant, *F*(2,274) = 2.06, *p* = 0.129, *d* = 0.24. Testing our *a priori* hypothesis with focused planned contrasts, we observed that participants in the *conformity MCII* condition gave more correct answers on the task (*M* = 2.57, *SD* = 1.50), compared with participants in both the *conformity RC* condition (*M* = 1.56, *SD* = 1.59), and the *conformity NSR* condition (*M* = 1.96, *SD* = 1.60), *t*(274) = 2.16, *p* = 0.032, *d* = 0.26 (Figure 4).

In sum, we replicated the previous findings. Importantly, these results highlight that it is not the provided informational content that leads to a regulation of one’s tendency to conform, but rather the way *how* people elaborate on that information (fantasy realization theory; Kappes et al., 2012; Kappes et al., 2013; review by Oettingen, 2012). Participants in the reverse contrasting condition thought about the same content as participants in the MCII condition and still conformed more often to the supposed majority. They also tended to give more incorrect answers compared to participants engaging in MCII. Again, the latter result needs to be interpreted with caution due to the interaction effect between conformity and the self-regulation condition not being significant.

However, one could argue that the observed patterns may be ascribed to demand characteristics, i.e., participants engaging in MCII were asked to follow *if-then* plans with pre-specified contents (i.e., “If I feel that I follow other people’s behavior, then I will tell myself: Ignore them!”). In Study 4, we therefore aimed to explore whether mental contrasting would help participants to *autonomously* generate *if-then* plans that support their regulation of conformity and their performance on the task.

## Study 4: MCII Entailing Idiosyncratic If-Then Plans

In Study 4, we hypothesized that participants in the conformity condition would choose the incorrect answers indicated by the supposed majority less often and correct answers more often when engaging in MCII (vs. no self-regulation), even when they generated idiosyncratic *if-then* plans.

### Method Study 4

#### Participants and Power Analysis

We recruited 200 participants. We excluded 23 participants because they indicated suspicion about the conformity manipulation (*n* = 17; for example, one participant wrote “I don’t think the MTurk stats were true.”) or did not follow the instructions of the manipulation of self-regulatory thought (*n* = 6; for example, one participant wrote “[If] I’m not able to lose weight by dieting [then] I plan on joining the gym with my brother. instead of an if-then plan). All participants were recruited on MTurk and received $2.00 for their participation. Our final sample consisted of 177 participants (57% women). Participants’ ages ranged between 18 and 82 years, with a mean of age of 35.33 years (*SD* = 12.13). Participants were randomly assigned to one of four conditions (see Table 1). A sensitivity power-analysis indicated that with 84 participants (within the two conformity conditions) and 93 participants (within the two control conditions) we would have 90% power to find, in each case, a medium to large effect (*d* = 0.72 and *d* = 0.68).

#### Procedure and Materials

The procedure and materials were identical to those used in the previous studies. However, in the present study, the logical reasoning task consisted of five test items and five practice items in order to familiarize participants with the logical reasoning task. Additionally, we adapted the MCII exercise in order to let participants formulate idiosyncratic if-then plans.

##### Logical reasoning task: practice phase

After participants were randomly assigned to either the conformity or the control conditions, we asked them to work on five logical reasoning items (*Standard Progressive Matrices*; Raven, 1965)^4^. Below the items, we presented the same diagrams used in the previous studies, claiming to show how other MTurk participants had answered in the past. Diagrams shown in the control condition displayed equally distributed answers. Diagrams shown in the conformity condition displayed the majority of participants choosing one specific answer. Specifically, for the conformity condition, there were two filler items (i.e., diagrams showed the majority of MTurk participants choosing the *correct* answer) and three critical items (i.e., diagrams showed the majority of MTurk participants choosing an *incorrect* answer).

##### MCII: idiosyncratic plans

In the MCII condition, we asked participants to name and elaborate on one positive aspect that they would associate with independently solving the upcoming logical reasoning task, followed by the obstacle that might hinder them from independently solving the logical reasoning task. Thereafter, instead of presenting participants a pre-specified *if-then* plan, we now asked participants to autonomously generate their own *if-then* plan, where they defined exactly when and how they wanted to act to achieve their goal. Specifically, all participants read: “Please make the following plan for yourself: If… (obstacle), then I will (action to overcome obstacle).” Participants had to first identify and fill in their obstacle in the if-part, which they specified predominantly in accordance with our expectations, i.e., participants named the perception of other MTurk workers answers’ or behaviors. They then had to identify and fill in an instrumental action in the then-part, which mostly referred to staying focused on one’s own behavior instead of getting distracted by other people’s answers ([If] I feel myself studying other’s answers, [then] I will redirect my attention to figuring out the problems myself”; “If I am following the behavior of other people, [then] I will think critically for myself”). Participants who did not name an if- or a then part were excluded from the analysis.

### Results and Discussion Study 4

#### Expectation, Incentive, and Commitment

Mean values for expectations [*M* = 5.13, *SD* = 1.42; *t*(176) = 10.60, *p* < 0.001], incentive value [*M* = 5.76, *SD* = 1.27; *t*(176) = 18.38, *p* < 0.001], and commitment [*M* = 4.30, *SD* = 1.70; *t*(176) = 2.35, *p* = 0.020] were above the midpoints of the scales and did not differ between conditions (all *p*’s > 0.5, see Supplementary Materials).

#### Conformity on the Logical Reasoning Task

All data reported in the following focus exclusively on the test phase of the logical reasoning task. Identical to the practice phase, participants could conform 0–3 times in the test phase. Importantly, those participants who were assigned to the conformity condition in the practice phase were also assigned to the conformity condition in the test phase.

We conducted a two-way ANOVA with conformity (*conformity* vs. *control*) and condition (*MCII* vs. *no self-regulation*) as between-subject factors and the number of conform answers on the task as dependent variable. There was a significant main effect of conformity, *F*(1,173) = 27.21, *p* < 0.001, *d* = 0.78, as well as a significant main effect of condition, *F*(1,173) = 4.23, *p* = 0.041, *d* = 0.31. The interaction between conformity and condition was also significant, *F*(1,173) = 14.36, *p* < 0.001, *d* = 0.58. We conducted focused planned contrasts to test our *a priori* hypotheses. Planned contrasts for the conditions adopting no self-regulatory thought (*conformity no self-regulation* vs. *control no self-regulation*) revealed that participants in the *conformity no self-regulation* condition chose the incorrect answers indicated by the supposed majority more often (*M* = 1.23, *SD* = 0.96) compared to participants in the *control no self-regulation* condition (*M* = 0.26, *SD* = 0.45), *t*(59.95) = 6.08, *p* < 0.001, *d* = 1.30.

To investigate our second hypothesis, i.e., the self-regulation of conformity, we conducted planned contrasts for the two conformity conditions (*conformity MCII* vs. *conformity no self-regulation*). Participants in the *conformity MCII* condition chose the incorrect answers indicated by the supposed majority less often (*M* = 0.60, *SD* = 0.81) compared with participants in the *conformity no self-regulation* condition (*M* = 1.23, *SD* = 0.96), *t*(81.55) = 3.24, *p* < 0.001, *d* = 0.71 (Figure 3).

#### Self-Regulation of Correct Behavior

Identical to Study 1 to 3, we performed our analysis of correct behavior on the three critical items. Thus, participants could obtain a maximum of three correct answers on the task. We conducted a two-way ANOVA with conformity (*conformity* vs. *control*) and condition (*MCII* vs. *NSR*) as between-subject factors and the number of correct answers on the task as dependent variable. There was a significant main effect of conformity, *F*(1,173) = 4.28, *p* = 0.040, *d* = 0.32, while there was, however, no significant main effect of condition, *F*(1,173) = 0.43, *p* = 0.515, *d* = 0.08. The interaction between conformity and condition was significant, *F*(1,173) = 5.04, *p* = 0.026, *d* = 0.34. Testing our *a priori* hypothesis, we conducted focused planned contrasts for the two conformity conditions (*conformity MCII* vs. *conformity no self-regulation*). Participants in the *conformity MCII* condition tended to give more correct answers on the task (*M* = 1.60, *SD* = 1.03), compared with participants in the *conformity no self-regulation* condition (*M* = 1.18, *SD* = 1.11), *t*(81.93) = 1.79, *p* = 0.077, *d* = 0.39 (Figure 4).

To sum up, we confirmed our hypothesis: Participants who engaged in MCII and generated idiosyncratic *if-then* plans chose the incorrect answers indicated by the supposed majority less often than participants who did not engage in a self-regulation strategy. Furthermore, those who engaged in MCII (vs. no self-regulation strategy) tended to give more correct answers on the task compared with participants who did not engage in a self-regulation strategy, although this difference did not reach significance. Importantly, our results show that the regulation of conforming behavior is not solely driven by a pre-specified *if-then* plan. Rather, when participants first elaborated on the desired future and subsequently on an obstacle in the present reality, they were able to generate effective *if-then* plans by themselves, which, in turn, helped them regulate their tendency to conform and to succeed in solving the task.

### Meta-Analysis: Correct Answers

Looking at the correct answers throughout all four conducted studies, we observed that in Studies 2 and 3, participants in the conformity condition provided significantly more correct answers on the task when engaging in MCII (vs. other conditions), though these results need to be taken with caution as the interaction effects between conformity and self-regulation condition were not significant; in Study 1 and 4, however, the planned contrasts results were only marginally significant. Interestingly, in Study 4, in which participants were permitted to find their own idiosyncratic if-then plan, the interaction effect between conformity and the self-regulation condition was significant.

**FIGURE 5:**
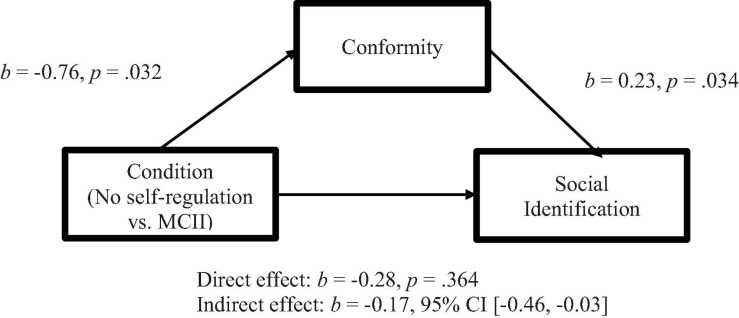
Model of condition (No self-regulation vs. MCII) as a predictor of social identification with the group of MTurk participants, mediated by conforming behavior during the task (Study 1). The confidence interval for the indirect effect is a bootstrapped CI based on 5000 samples.

To account for the inconsistent pattern of findings across the four studies reported in the present paper, we conducted a meta-analysis consulting the MAVIS Meta-Analysis via Shiny software (Version 2.1; Hamilton et al., 2014), to assess the general effect size of our manipulation on the dependent variable of correct answers. We used a random effects model to analyze all four studies. The test for heterogeneity revealed that the effect sizes did not significantly differ between the four studies (I^2^ = 0%). Across Studies 1 to 4, the overall effect size of MCII on the number of correct answers in the logical reasoning task was *Hedges’s g* = 0.28 [0.11, 0.46] based on *k* = 4 involving 789 participants (Figure 5). Accordingly, we assume that MCII (vs. other conditions) might help participants to improve their performance on the logical reasoning task by increasing the number of correct answers despite a deviant majority.

## General Discussion

In four experimental studies, we investigated whether the self-regulation strategy of MCII is an effective tool to regulate people’s tendency to conform to a majority. We introduced a computer-based logical reasoning task, in which we reliably induced conformity. Using this task, we showed that MCII (vs. relevant control conditions) helped people to effectively self-regulate their tendency to conform in the face of deviant majority influence. We showed this pattern of findings comparing MCII to a no self-regulation control condition (Studies 1, 2, and 4) and to an active reverse contrasting control condition (Study 3). We observed our findings with MCII including both pre-specified *if-then* plans (Studies 1 to 3), and idiosyncratic *if-then* plans (Study 4). Importantly, MCII (vs. relevant control conditions) seemed to help people attain their goal of independently solving the task, as indicated by increases in correct answers in the task. The latter findings, however, should be interpreted with caution as in three of the four studies the interaction effects of conformity and the self-regulation condition were non-significant.

### Self-Regulation of Conformity

Regarding our findings on conformity, we want to discuss three points: First, one may argue that participants in the MCII condition (vs. other) obtained more relevant information (i.e., we told them “People tend to follow other people’s behavior when they are unsure of how to act”), facilitating identification of the obstacle and subsequently the regulation of one’s tendency to conform. Study 3 refutes this alternative explanation. We utilized the control condition of reverse contrasting, whereby participants elaborated on exactly the same outcome and obstacle as participants in the MCII condition, but in a different order and followed by mute if-then plans (i.e., “If I solve all following tasks successfully, then I will feel great!”). However, only when participants engaged in MCII did they reduce the number of conform answers.

Second, one may argue that participants engaging in MCII simply followed the instruction of the experimenter to ignore the majority’s behavior (i.e., “If I feel that I follow other people’s behavior, then I will tell myself: ‘Ignore them!”’), and as such conformed to the experimenter’s request instead of the majority influence. The pattern we observed across four studies refutes this notion: Participants did not simply choose any answer other than the one allegedly given by the majority. Instead of blindly following the experimenter’s instructions to ignore other people’s behavior, participants who used MCII seemed to have gotten it right more often as they tended to choose more often the correct option among eight choices. Additionally, in Study 4, participants generated *idiosyncratic if-then* plans which, similarly to the pre-specified plans in Studies 1, 2, and 3, should have helped them to regulate their conform behavior and to improve their performance on the task. Indeed, it was in this last study that the interaction effect between conformity and self-regulation condition regarding finding the correct answer was significant. Thus, it seems unlikely that the instructions given in the pre-specified plans of Studies 1, 2, and 3 would have caused the observed effects. Future studies should replicate the present findings by letting people formulate idiosyncratic obstacles and idiosyncratic *if-then* plans, instead of prompting them with the obstacle of following other people’s behavior and giving them the plan to ignore other people’s behavior. Further, future research might assess reaction times in addition to participants’ responses as an indicator of effort, to gain stronger evidence that MCII leads people to spend effort on pursuing their goal to perform well rather than blindly following the experimenter’s request to ignore the majority.

Third, one may argue that the strategy was very easy to apply in a context, where people do not have to fear real world consequences (e.g., exclusion from a group) when behaving deviant. However, people do not only conform because of real but also because of imagined social pressure (e.g., Deutsch and Gerard, 1955; Park and Feinberg, 2010). Accordingly, participants in our studies might have feared that they might look foolish in the eyes of imagined others (e.g., their collaborators, family, and friends). Such imagined embarrassment might even result in a diminished subjective sense of competence or efficacy (Bandura, 1977). Additionally, though it can be hardly assumed that being a MTurk worker is the central identity of our participants (Buhrmester et al., 2011), they still might have imagined the shame of feeling responsible for a possible negative outcome of their in-group, that is the MTurk workers vs. the bankers. Future research should investigate whether MCII can foster the self-regulation of conformity more readily when people’s in-group is central to their identity. Specifically, the tendency to conform should increase when a person identifies with the group. And because MCII has been shown to be particularly effective when challenges are high (Wittleder et al., 2019; Mutter et al., 2020), we would expect MCII to be more effective the stronger people identify with their in-group.

### Correct Performance

Regarding correct performance, three points might elucidate the tentative observation that self-regulation by MCII supported participants in the conformity condition to increase the number of correct answers. First, MCII geared toward *ignoring other people’s behavior* should be effective in facilitating correct answers. Motivating people to ignore other people’s behavior may free cognitive capacities for the task. Instead of spending time to question other MTurker’s answers, participants could now specifically focus on the task itself. However, this task focus should only appear in the MCII condition, that is, when the best outcome is elaborated first and the obstacle is elaborated afterward (Kappes et al., 2012, 2013). As results of Study 3 seem to suggest, participants adopting a reverse order of elaboration (i.e., reverse contrasting) were not as successful in solving the task as participants adopting MCII.

Second, the question may arise what conformity means for participants in the control condition since there is no prominent answer chosen by a majority, which they could follow or avoid. However, people generally tend to look at other people’s judgments when being insecure (e.g., Festinger, 1954; Deutsch and Gerard, 1955; Erb and Bohner, 2007). Participants in the present four studies were not instructed to avoid conformity *per se* but rather to ignore other people’s behavior and, by this, to exclusively focus on their own performance. Thus, the goal to avoid conformity should not have preoccupied participants’ mind – neither in the conformity nor the control conditions. The focus on their own performance was evident in the positive outcomes that participants associated with attaining their goal (participants predominantly imagined their feelings of pride, control, or confidence).

A third point to be discussed is the impact of MCII (vs. no self-regulation/reverse contrasting) on the number of correct responses in the no-conformity control conditions. In all four studies, MCII vs. no self-regulation/reverse contrasting did not significantly differ in the control conditions (though in Studies 1 and 2, MCII appeared to additionally increase the number of correct responses). Explaining the non-significant differences in correct answers between MCII and the no self-regulation/reverse contrasting conditions, we speculate that participants using no self-regulation strategy or reverse contrasting could exclusively focus on finding the correct answers. On the contrary, participants adopting MCII now may have started to check other people’s answers for additional information which might have diverted their attention. This diversion of attention should have resulted in relatively fewer correct answers. In addition, by adopting MCII, participants were made aware of the potential obstacle. However, when this obstacle did not appear later, participants in the MCII condition had no opportunity of overcoming their obstacle and thus no opportunity of performing better than those in the no-self-regulation or reverse contrasting condition.

### Implications for Research on Conformity and on MCII

The present research adds to the literature on conformity, presenting an individually applicable self-regulation strategy that helps people to effectively regulate their tendency to conform and that might be applicable across various situations in which conforming to others may be disadvantageous to their goal pursuit. In addition, the findings of our studies may seem rather artificial when it comes to directly applying them to pressures to conform in daily life. Therefore, we need field studies to understand under which circumstances and for whom MCII can best help to regulate conformity behavior during everyday life.

Next to pursuing one’s own goals and solving one’s own problems rather than focusing on possible disadvantages of deviating from others (e.g., Asch, 1955; Rosander and Eriksson, 2012), such situations may include focusing on one’s own skills and abilities when solving a task, engaging in independent consumer decisions (e.g., Gardner and Steinberg, 2005; Lee et al., 2008; Park and Feinberg, 2010), or resisting social influence to avoid risky decision making (e.g., Santor et al., 2000; Zhou et al., 2009). Future research should conceptually replicate the present findings in other domains in which the tendency to conform to other people’s behavior similarly hinders one’s individual goal pursuit.

Whereas in the present research we explicitly focused on situations where the tendency to act in a conform way hampers people by preventing the successful attainment of their individual goals, there are various situations in which conformity has beneficial consequences for the individual (e.g., Argyle, 1957; Hollander, 1958; Brewer, 1991; Bond and Smith, 1996). Therefore, the aim of the present study was not to find a strategy that excludes conformity *per se* from people’s daily behavior. Rather, MCII should help people to be more sensitive to potentially positive or negative social influence. By elaborating the most positive outcome and afterward the obstacle holding one back from attaining one’s goal, people should recognize when this obstacle is a majority opinion or something else such as laziness^5^.

In line with this differentiated view of the consequences of conformity, it is not the strategy of MCII *per se* that reduces conformity with the majority group. Rather, MCII is a content-independent strategy, meaning that it regulates goal pursuit applied to a wide range of topics and life domains (e.g., interpersonal, achievement, health; review by Oettingen, 2012). It can be adopted for any wish one wants to realize and therefore acts in the service of the content to which it is applied. To illustrate the versatility of the strategy, one could also adopt MCII to *increase* one’s conformity to the members of the majority group. For example, one could define establishing a close relationship with other group members as a wish or goal, in which case non-conforming feelings and actions would be the obstacle standing in the way of attaining that goal. In this scenario, people engaging in MCII (vs. relevant control conditions) would be hypothesized to increase their conformity with the members of the group.

The present research also adds to the literature on MCII, pointing toward the strategy’s effectiveness to attain one’s goals despite obstacles in the interpersonal domain. Whereas previous studies on MCII in the interpersonal domain primarily focused on the improvement of relationships and interactions between two individuals (e.g., Houssais et al., 2013; Kirk et al., 2013), in the present research MCII was used to support people in *detaching* from the social influence elicited by others in a situation in which this influence distracted people from attaining their personal goals. Future studies should elucidate the processes that render MCII effective for regulating conformity. For example, strong associative links between the obstacle and the behavior to overcome the obstacle may be implicated. These processes have already been shown in rendering MCII effective in other life domains such as the interpersonal and the health domains (see Kappes et al., 2012; Kappes and Oettingen, 2014).

### Conformity in a Computer-Mediated Context

Even though conformity is primarily known as a consequence of physical interaction, a range of studies showed that it also occurs in computer-mediated contexts (e.g., Bargh and McKenna, 2004; Cinnirella and Green, 2007; Park and Feinberg, 2010; Rosander and Eriksson, 2012). In the present research, we made social identity salient by introducing the community of MTurk participants as an in-group, whose results were allegedly compared to those of an out-group (i.e., bankers). Even though social identity with the group and, by this, the fear of being deviant may have led participants to conform to the group (*normative influence;* Deutsch and Gerard, 1955), insecurity about the correct answers might have also led participants to conform (*informational influence;* Deutsch and Gerard, 1955). However, it was neither the focus of the present paper to investigate, whether it is normative or informational influence causing participants to conform nor to investigate whether it specifically is the ingroup norm that causes conformity. We rather included social identity as an additional possibility for strengthening the circumstances under which conformity generally occurs. Future research should investigate the reasons underlying participants’ motivation to (non-)conform in computer mediated contexts.

One might argue that our instruction might have induced an intergroup situation in which the ingroup (MTurkers) might be perceived as less capable as the outgroup (bankers). This might have undermined the credibility of MTurk response choices in the conformity condition. However, we assume that our presentation of the logical reasoning task could have motivated our MTurk participants to solve the task correctly, since we depicted them as “people who occasionally deal with social psychology experiments.” They might have inferred that our experiment was a social psychology experiment, and thus that our task was a psychology task rather than an economic problem which would be more familiar to bankers than to them.

## Conclusion

Thinking back to the example in the beginning of this paper, our results suggest that MCII will help you decide whether you would let your friends choose the answer in the last round of the quiz or whether you would stick to your answer and speak up. By using MCII, you should become more aware of the obstacle holding you back from attaining your goal, for example, your urge to conform to your friends. Thus, you could identify and engage in appropriate behaviors to overcome that obstacle and achieve your goal of finding the correct solution to the tricky problem.

## Data Availability Statement

The raw data supporting the conclusions of this article will be made available by the authors, without undue reservation.

## Ethics Statement

The studies involving human participants were reviewed and approved by University of Hamburg 75 2016 KA Riess Schlussvotum. The patients/participants provided their written informed consent to participate in this study.

## Author Contributions

VW conducted the studies. NK and VW analyzed the data. All the authors contributed to writing the manuscript.

## Conflict of Interest

The authors declare that the research was conducted in the absence of any commercial or financial relationships that could be construed as a potential conflict of interest.

## Footnotes

1 Participants did not receive feedback concerning their performance in any of the studies.

2 Participants were excluded since we wanted to make sure that we investigated the pure mechanisms of conformity and its regulation, independently from any suggestions concerning the purpose of the study. However, even when we included all participants in the analysis, there was still a significant difference for the number of conform answers between participants in the control and conformity condition. Also, we still found the significant effect that MCII (vs. no self-regulation or reverse contrasting) helps to regulate conformity.

3 With regard to the filler items, there were no significant differences concerning the number of correct answers between participants in the *conformity no self-regulation* condition and the *conformity MCII* condition in all studies (all *p*’s >0.05).

4 Participants worked on 10 logical reasoning items in total (five items in the introduction phase and five items in the experimental phase); eight of the items were already used in Studies 1 to 3. Thus, we added two new items from the *Standard Progressive Matrices* (Raven, 1965), which were of equal medium difficulty.

5 As mentioned earlier, there was no significant difference regarding the number of correct answers between participants adopting MCII or reverse contrasting/no self-regulation. This implies that participants did not simply ignore the majority’s answers but rather focused on their own judgment, independent of whether it is same or different to that of the majority.
