# Supplementary material for: The Self-Regulation of Conformity: Mental Contrasting With Implementation Intentions (MCII)
Source: Front Psychol. 2021 Jun 2;12:546178. doi: 10.3389/fpsyg.2021.546178 (PMC8206508; doi:10.3389/fpsyg.2021.546178)
Supplement: Supplementary file 1 [file Table_1.docx]

**Pilot Study**

**Introduction**

In the present research, we developed a computer-based paradigm based on an experimental design by Rosander and Eriksson (2012) investigating conforming behavior in the context of computer-mediated communication (CMC; e.g., Bak & Kessler, 2012; Bargh & McKenna, 2004; McKenna & Bargh, 1998; Riva, 2002; Williams, Cheung, & Choi, 2000). While some studies have shown that the effects of conformity appear to diminish in the context of CMC (compared to face-to-face interaction) due to deindividuation (i.e., feeling of being indistinguishable from others; Cinnirella & Green, 2007; McKenna & Green, 2002; Smilowitz, Compton, & Flint, 1988; Zimbardo, 1969), other studies, have found that deindividuation can lead to enhanced conformity within the context of CMC. According to the SIDE model (Social Identity of Deindividuation; Spears & Lea, 1992), an anonymous situation increases the salience of a group identity *if* one is available, while the norms connected to that group’s identity govern the individual’s behavior, subsequently leading to conformity (Postmes & Spears, 2002; Postmes, Spears, & Lea, 2002; Rogers & Lea, 2005; Rosander & Eriksson, 2012).

**Method Pilot Study**

**Participants**

Based on the study by Rosander and Eriksson (2012), we assumed a medium-to-large effect size (*d =* 0.73), which we applied to an a priori power analysis for two conditions within a t-test. The power analysis indicated that approximately 66 participants would be needed to achieve 90% power (1 - *β*) at a .05 alpha level (α = .05). Sixty one participants were recruited on MTurk (50.8% women). Participants received $1.50 for their participation. Participants were between 20 and 74 years old (*M* = 37.56, *SD* = 14.21). Participants were randomly assigned to either the conformity condition (*n* = 31), or the control condition (*n* = 30). There were no significant differences between the conditions regarding participants’ age, *t*(59) = 1.15, *p* = .254, *d* = 0.31 or gender, $\chi$^2^ = 0.02, *p* = .548, Cramer’s *V* = .017.

**Procedure and Materials**

All participants completed the study online. Prior to their participation, they were informed about the procedure of the study and completed the consent form. After reading the cover story, participants started working on the eight logical reasoning items and were finally asked for some demographic data.

**Social identification.** To implement a feeling of shared social identity with the majority – in this case the group of MTurk participants – we presented participants a cover story indicating that the survey was designed to compare cognitive abilities of people who occasionally deal with social psychology experiments (e.g., MTurk participants) and people who deal with economic problems only (e.g., bankers).

We used three items with a Likert scale from 1 (*not at all*) to 7 (*very much*); i.e., “To what extent do you feel as part of the group of MTurk participants?”, “To what extent do you identify yourself with the group of MTurk participants?”, and “How important is it to you that your group obtains a good overall result?” The three items were combined into one scale (α_t1_ = .83). After the logical reasoning task, the three items were repeated to investigate whether social identification with the group of MTurk participants changed during the experiment (α_t2_ = .88). High scores on the scale indicate a strong social identification with the group of MTurk participants.

**Logical reasoning task.** The logical reasoning task consisted of eight logical items, which were chosen from Standard Progressive Matrices (Raven, 1965). For each item, there were eight answer options, of which only one was the correct one. For both the control and conformity groups, we presented diagrams below each of the logical reasoning items, pretending to show the answers other MTurk participants had given in the past. While diagrams in the control condition showed equally distributed answers (Figure 1), diagrams in the conformity condition showed one out of the eight options that was more frequently chosen, thus representing a majority answer (Figure 2)^^[[1]](#footnote-1)^^.

In the conformity condition, five of the eight items revealed diagrams representing a majority of MTurk participants choosing an *incorrect* answer (i.e., critical items). We defined the majority as a larger portion of the group; with the five items each chosen by 48%, 58%, 59%, 72% and 76% of MTurk participants, respectively. The minority answers were distributed to the other seven answer options (each ranging between 0% and 8%). In order to avoid suspicion of a conformity manipulation (Stang, 1976), we included three items which revealed diagrams representing a majority of MTurk participants choosing the *correct* answer (i.e., filler items). For all our studies, we presented the eight items in order of increasing difficulty (i.e., ranging from easy to difficult).

**Results Pilot Study**

Identical to Rosander and Eriksson (2012), we only included the five critical items in the analysis. Accordingly, participants in the conformity condition were able to conform zero to five times. We quantified conformity as the difference between the number of answers that agreed with the supposed majority answers for participants in the conformity condition and the number of the same incorrect answers for participants in the control condition.

For the five critical items, we found that participants in the conformity condition chose the incorrect answer indicated by the supposed majority significantly more often (*M* = 2.42, *SD* = 1.65), compared to participants in the control condition (*M* = 0.70, *SD* = 0.95), *t*(59) = 4.97, *p* < .001, 95 % CI [-2.41, -1.03], *d* = 1.28. Thus, we successfully induced conformity in the computer-based paradigm.

**Study 1**

**Social Identification**

To strengthen the cover story and to highlight participants’ shared social identity to the group of MTurk workers, we used the same three items as in the pilot study, pointing at the shared social identity as well as the common goal all MTurk workers should have (e.g.”To what extent do you feel as part of the group of MTurk participants?”) . The three items were combined into one scale (α_t1_ = .83).

We aimed to examine a possible reciprocal relation of social identification with the majority (i.e., the group of MTurk participants) and conformity within our paradigm. According to the SIDE model, social identification with the majority (i.e., the group of MTurk participants) should predict conformity on the task. Conforming to the majority’s behavior, in turn, may predict higher social identification with the majority afterwards. Thus, we assumed that the effect of condition (MCII vs. no self-regulation strategy) on social identification with the source of influence should be mediated by conforming behavior (Figure 6, supplemental materials).

**Results Study 1**

**Expectations, incentive, and commitment**

Mean values for expectations (*M* = 5.12, *SD* = 1.49), incentive value (*M* = 6.07, *SD* = 1.10) and for commitment (*M* = 4.84, *SD* = 1.68) were above the midpoints of the scales. There were no significant differences between the conditions regarding the items (expectations *F*(3, 133) = .868, *p* = .460, *d* = 0.28; incentive *F*(3, 133) = .411, *p* = .746, *d* = 0.18; commitment *F*(3, 133) = .422, *p* = .738, *d* = 0.18).

**Social Identification and Conformity**

We first compared participants’ self-reported social identification with the group of MTurkers between both conformity conditions after the logical reasoning task. There was a marginally significant difference between the *conformity MCII* and the *conformity NSR* condition: Those who engaged in MCII identified less with the group (*M* = 4.28, *SD* = 1.49) compared to those who did not engage in the self-regulation strategy (*M* = 5.02, *SD* = 1.59), *t*(64) = 1.94, *p* = .056, 95 % CI [-0.02, 1.50], *d* = 0.48.

Further, we investigated the relation between social identification and conformity. We found a marginally significant positive correlation between social identification assessed at Time 1 and the number of conform answers, *r*(66) = .22, *p* = .07, 95 % CI [0.01, 0.42], as well as a significant positive correlation between the number of conform answers and social identification assessed at Time 2, *r*(66) = .45, *p* < .001, 95 % CI [0.24, 0.62]. The more participants identified themselves with the group, the more they acted conform, and the more they acted conform, the more they identified themselves with the group afterwards. Correlations for both conformity groups did not significantly differ from each other (Time 1: *conformity MCII* *r*(31) = .17, *p* = .352, 95% CI [-0.07, 0.42], *conformity NSR* *r*(35) = .23, *p* = .183, 95% CI [-0.08, 0.50], *z* = .242, *p* = .405; Time 2: *conformity MCII* *r*(31) = .39, *p* = .030, 95% CI [0.11, 0.64], *conformity NSR* *r*(35) = .43, *p* = .011, 95% CI [0.14, 0.67], *z* = .186, *p* = .426).

Lastly, we tested whether the effect of self-regulation condition (*conformity MCII* vs. *conformity NSR*) on social identification was mediated by conforming behavior on the task. To do so, we first calculated a change score for social identification by subtracting Time 1 from Time 2; the more negative the value, the more the social identification decreased over the task. We followed a bootstrapping procedure using the SPSS PROCESS macro provided by Hayes (2013). The indirect effect of condition on change of social identification through conforming behavior in the logical reasoning task was significantly different from 0, 95% CI [-0.45, -0.03] with 5000 iterations (Figure 6). That is, people who engaged in MCII (vs. no self-regulation strategy) conformed less to the bogus majority answers, which subsequently led them to identify less with the majority group.

**Discussion Study 1**

We found a positive relation between the identification with the group of MTurk participants and conforming behavior during the task. This finding is in line with previous research indicating that social identification with the source of influence is an important determinant of conforming behavior (e.g., Cinnirella & Green, 2007; David & Turner, 1996; Postmes & Spears, 2002; Rogers & Lea, 2005). Moreover, the effect of condition (MCII vs. no self-regulation strategy) on change of social identification was mediated by conforming behavior during the task. That is, participants who engaged in MCII (vs. no self-regulation strategy) conformed less to the supposed majority, which led them to identify less with the group of MTurk participants.

**Results Study 2**

**Expectations, incentive, and commitment**

Mean values for expectations (*M* = 5.27, *SD* = 1.38), incentive (*M* = 6.08, *SD* = 1.03) and commitment (*M* = 4.64, *SD* = 1.60) were above the scale midpoints. There were no significant differences in expectations, incentive, or commitment between the four conditions (expectations *F*(3, 191) = .556, *p* = .645, *d* = 0.18; incentive *F*(3, 191) = .156, *p* = .926, *d* = 0.10; commitment *F*(3, 191) = .622, *p* = .602, *d* = 0.18).

**Social Identification and Conformity**

Similar to Study 1, participants in the *conformity MCII* condition identified less with the group of MTurk participants after the logical reasoning task (*M* = 4.11, *SD* = 1.44) compared to participants in the *conformity NSR* condition (*M* = 4.83, *SD* = 1.28), *t*(94) = 2.61, *p* = .011, 95 % CI [0.17, 1.28], *d* = 0.53.

Investigating the relation between social identification and conformity, we found a significant positive correlation between conformity and social identification with the group assessed at Time 2, *r*(96) = .47, *p* < .001, CI [0.32, 0.61]. Correlations did not differ between both conformity conditions (*conformity MCII* *r*(46) = .45, *p* = .002, 95% CI [0.18, 0.67]; *conformity NSR* *r*(50) = .44, *p* = .001, 95% CI [0.18, 0.63]; *z* = -.059, *p* = .476).

To test whether the effect of condition (MCII vs. no self-regulation strategy) on social identification was mediated by conforming behavior, we calculated a change score for social identification by subtracting Time 1 from Time 2 and included this as the dependent variable in a bootstrapping procedure using the SPSS PROCESS macro provided by Hayes (2013). As in Study 1, the indirect effect of condition on change of social identification through conforming behavior was significantly different from 0, 95% CI [-0.461, -0.047] with 5000 iterations. That is, people who engaged in MCII (vs. no self-regulation strategy) conformed less to the supposed majority answers, which subsequently reduced their social identification with the majority.

**Discussion Study 2**

We confirmed the close connection of conformity and social identification on the task: The effect of condition (MCII vs. no self-regulation strategy) on social identification was mediated by conformity in the task.

**Results Study 3**

**Expectations, incentive, and commitment**

Mean values for expectations (*M* = 4.99, *SD* = 1.40), incentive value (*M* = 5.85, *SD* = 1.22) and commitment (*M* = 4.43, *SD* = 1.02) were above the scale midpoints. There were no significant differences between the conditions concerning these items (expectations *F*(5, 274) = .582, *p* = .714, *d* = 0.20; incentive *F*(5, 274) = .729, *p* = .603, *d* = 0.22; commitment *F*(5, 274) = .588, *p* = .709, *d* = 0.20).

**Social Identification and Conformity**

There was no significant difference between the three conformity conditions regarding the social identification with the group of MTurk participants assessed at Time 2.

Investigating the relations between social identification and conformity, we found a significant positive correlation between social identification assessed at Time 1 and conforming behavior on the task, *r*(142) = .17, *p* = .041, 95 % CI [0.03, 0.30], as well as a significant positive correlation between conforming behavior on the task and social identification assessed at Time 2, *r*(142) = .39, *p* < .001, 95 % CI [0.25, 0.51]. Correlations did not significantly differ between the three conditions (Time 1: c*onformity* *NSR* *r*(53) = .12, *p* = .387, 95% CI [-0.11, 0.37], c*onformity* *MCII r*(44) = .25, *p* = .096, 95% CI [0.03, 0.49], c*onformity RC r*(45) = .22, *p* = .152, 95% CI [-0.05, 0.46]; Time 2: co*nformity* *NSR* *r*(53) = .28, *p* = .040, 95% CI [0.01, 0.52], c*onformity* *MCII r*(44) = .52, *p* < .001, 95% CI [0.31, 0.72], c*onformity RC r*(45) = .46, *p* = .002, 95% CI [0.25, 0.63]).

Next, we tested whether the effect of condition (MCII vs. other) on change of social identification was mediated by conforming behavior on the task. We observed that the indirect effect of condition on change of social identification through conforming behavior was significantly different from 0, 95% CI [-0.391, -0.087], with 5000 iterations. Participants engaging in MCII (vs. no self-regulation and reverse contrasting) conformed less to the supposed majority answers, which subsequently reduced their social identification with the majority.

**Discussion Study 3**

We confirmed the close connection of conformity and social identification on the task: The effect of condition (MCII vs. other) on social identification was mediated by conformity in the task.

**Study 4**

**Expectation, incentive, and commitment**

Mean values for expectations (*M* = 5.13, *SD* = 1.42), incentive value (*M* = 5.76, *SD* = 1.27) and commitment (*M* = 4.30, *SD* = 1.70) were relatively high and did not differ between conditions (expectations *F*(3, 173) = .655, *p* = .581, *d* = 0.22; incentive *F*(3, 173) = .505, *p* = .215, *d* = 0.32; commitment *F*(3, 173) = .705, *p* = .168, *d* = 0.34).

**General Discussion**

In three studies, we observed that the effect of self-regulatory thought (MCII vs. other) on social identification with the majority (i.e., the group of MTurk participants) was mediated by conforming behavior in the logical reasoning task.

**Social Identification and Conformity**

Perceived similarity with the source of influence is a determining factor for conformity (e.g., David & Turner, 1996). Only a little perceived similarity with another person (e.g., shared names or birthdays) may lead to enhanced conformity or compliance (Burger, Soroka, Gonzago, Murphy, & Somervell, 2001; Cialdini & Goldstein, 2004). Our results are in line with this argument: Participants socially identified with the group of MTurk participants. In fact, the more participants identified with the group of MTurk participants, the more likely they were to conform to the behaviors of their MTurk peers.

Studies 1 to 3 revealed another interesting finding, which is, to our knowledge, only scarcely discussed in the existing literature: The relation between conformity and subsequent social identification with the source of influence. Despite the lack of previous discussion, the relationship between these variables is not entirely surprising, since being in line with a group’s behavior can enhance the perception of similarity, which in turn may further support the adoption of the group’s identity (see also Turner, 1991). Such reciprocal relationships have been described in other areas of psychological research. In his model of reciprocal determinism, for example, Bandura (1978) found that strong past performance predicts high self-efficacy beliefs, which in turn predict high subsequent performance. That is, people who feel efficacious regarding a certain behavior perform better, and this performance, in turn, strengthens their sense of efficacy. Results of Studies 1 to 3 show a similar reciprocal relationship between social identification and conforming behavior: The more participants socially identified with the majority, the more they conformed to the majority, and this conforming behavior, in turn, predicted social identification with the majority.

**MCII, Social Identification, and Conformity**

Participants who used MCII (vs. other) engaged less in conforming behavior and subsequently identified less with the source of influence (indirect effect). The effect of condition on change of social identification may suggest that MCII leads people to reinterpret the group’s performance in a way that results in less conformity. That is, participants in the MCII condition might have not perceived the majority’s answers as a potential source of information anymore, but rather as a distracting factor swaying their own behavior. Future studies should shed light on this hypothesis.

Importantly, while our studies focused on MCII as a strategy to reduce conformity and, in turn, social identification with the majority, it should be mentioned that, in general, our group memberships are an important factor that shape our (social) identity and that a reduction in social identification might not always be helpful for the individual. Since MCII is a content-independent strategy that can be applied to any wishes and concerns an individual might have, it could be easily applied to situations in which an individual has the wish to *strengthen* his or her identification with the group. Also here, MCII should help the individual to recognize obstacles standing in the way of realizing his or her wish and, in turn, help to overcome this obstacle and feel more connected to the social group. Future research should shed light on this hypothesis.

Another important aspect is the induction of conforming behavior in a computer-based context. Indeed, the successful induction of conforming behavior within our paradigm is in line with previous findings testing the SIDE model, indicating that intra- and inter-group processes are more powerful in the context of CMC than in face-to-face interactions *if* a strong sense of group identity is present (e.g., Cinnirella & Green, 2007; Laporte et al., 2010; Lee, 2006; Postmes & Spears, 2002; Rosander & Eriksson, 2012; Spears & Lea, 1992).

Even though MTurk participants are a highly diverse group (Buhrmester, Kwang, & Gosling, 2011), we assumed that CMC within our paradigm would reduce participants’ in-group heterogeneity and instead emphasize intragroup similarity. Indeed, our participants socially identified with the group of MTurk participants, and the more participants self-reported to identify with the in-group, the more they conformed to the group members’ judgments.

**References**

Bandura, A. (1977). Self-efficacy: Toward a unifying theory of behavioral change. *Psychological Review, 84,* 191–215. doi:10.1016/0146-6402(78)90002-4

Hayes, A. F. (2013). *Introduction to mediation, moderation, and conditional process analysis: A regression-based approach*. New York: Guilford Press.

McKenna, K. Y., & Bargh, J. A. (1998). Coming out in the age of the Internet: Identity “demarginalization“ through virtual group participation. *Journal of Personality and Social Psychology*, *75*, 681–694.

Postmes, T., Spears, R., & Lea, M. (2002). Intergroup differentiation in computer-mediated communication: Effects of depersonalization. *Group Dynamics: Theory, Research, and Practice*, *6*, 3–16. doi:10.1037/1089-2699.6.1.3

Smilowitz, M., Chad Compton, D., & Flint, L. (1988). The effects of computer mediated communication on an individual’s judgment: A study based on the methods of Asch’s social influence experiment. *Computers in Human Behavior*, *4*, 311–321. doi:10.1016/0747-5632(88)90003-9

Spears, R., & Lea, M. (1992). Social influence and the influence of the ’social’ in computer-mediated communication*.* In M. Lea (Ed.), *Contexts of computer-mediated communication* (pp. 30–65). New York, NY: Harvester-Wheatsheaf.

Spears, R., Postmes, T., Lea, M., & Wolbert, A. (2002). When are net effects gross products? Communication. *Journal of Social Issues*, *58*, 91–107. doi:10.1111/1540-4560.00250

1. Answers represented in the diagrams are shown in percentages. Accordingly, all answer options sum up to 100% in both control and conformity conditions. [↑](#footnote-ref-1)
